# Individual values, the social determinants of health, and flourishing among medical, physician assistant, and nurse practitioner students

**DOI:** 10.1371/journal.pone.0308884

**Published:** 2024-09-27

**Authors:** Stephanie Neary, Benjamin Doolittle, Martina Mueller, Michelle Nichols

**Affiliations:** 1 Department of Internal Medicine, Section of General Internal Medicine, Physician Assistant Online Program, Yale University, New Haven, Connecticut, United States of America; 2 Department of Internal Medicine, Section of General Internal Medicine, Yale University, New Haven, Connecticut, United States of America; 3 College of Nursing, Medical University of South Carolina, Charleston, South Carolina, United States of America; 4 Department of Public Health Sciences, Medical University of South Carolina, Charleston, South Carolina, United States of America; The Open University of Israel, ISRAEL

## Abstract

The purpose of this study is to explore how demographics and individual values, qualities, and personality traits are associated with perceptions of flourishing among medical (MD), physician assistant (PA), and nurse practitioner (NP) students. Current MD, PA, and NP students from two academic medical centers were recruited to participate in this cross-sectional study between August 6 and October 9, 2023. Participants completed the Secure Flourish Index (traditional SFI) and then applied a percentage weight to each of the six flourishing domains based on perceived relative importance to their overall flourishing. Additional survey questions included demographics and multiple validated instruments: WellRx, 2 Question Maslach Burnout Inventory, Brief COPE Inventory, the Short Grit Scale, and Duke University Religion Index. Descriptive statistics, ANOVA, correlation, and regression analyses were performed with an alpha of 0.05. A total of 393 of 1820 eligible students began the survey (21.6%) while 280, (15.4%) were included in the analysis. Traditional SFI scores were higher with higher grit (r = .368, p < .001). Traditional SFI scores were lower with higher WellRx (r = -.336, p < .001), burnout (r = -.466, p < .001), or avoidant (r = -.453, p < .001) coping style. Scores were about 10 points lower for students who had considered leaving training in the past 6 months (M = 75.3, SD = 16.2) than those who had not (M = 85.6, SD = 14.4; p < .001). The SFI domain of physical and mental health had the highest relative percentage weight (20.2% (SD 8.4)) but was second to lowest in mean domain flourishing score (mean 6.5, SD 1.7). While participants placed high value on physical and mental health, they reported relatively low flourishing in this area. Targeted interventions to improve the ability for students to cope with the hardships of training and life, as well as supports structured to address the social and structural determinants of health may improve flourishing among students with similar values.

## Introduction

Decreased mental health has been cited as a leading cause of medical and nursing student attrition and an estimated nearly 40% of students are experiencing burnout [1–3]. There are multiple intrinsic factors associated with student academic success, such as mental health, emotional coping, and resilience [4–6]. In addition, there are extrinsic factors, such as social determinants of health (SDOH) which make success difficult when unmet. The SDOH are the conditions in an individual’s environment that affect a broad range of outcomes including individual health and quality of life [7]. Healthy People 2030 categorizes the SDOH as economic stability, education access and quality, health care access and quality, neighborhood and built environment, and social and community context [7]. The longevity of medical and nursing training often requires short-term sacrifices for future gain and having passion and perseverance towards long-term goals has been shown to predict well-being [8].

Historically, wellness research has largely been focused on mitigating ill-being rather than on the promotion of well-being, which can be characterized by flourishing [9]. Flourishing encompasses a broad range of states ranging from hedonic measures of well-being like happiness to individual qualities such as virtue. In 2017, VanderWeele et al proposed that the determinants of flourishing could be characterized into six key domains: happiness and life satisfaction, physical and mental health, meaning and purpose, character and virtue, close social relationships, financial and material stability [9].

However, it has been found that individual perceptions of which domain is most important may vary by demographic factors and are likely influenced by cultural practices and beliefs [10]. Additionally, little is known if the importance of these domains changes based on life circumstances, such as pursuing graduate training, or outside factors, such as economic hardship. Individuals may knowingly reduce flourishing in one domain for improvement in another, which could occur by foregoing in-the-moment happiness for long term health [10]. A similar practice has been found among medical students who acknowledged trading personal mental or physical health for academic success [11]. These findings suggest that equally weighting each flourishing domain during scoring may not account for the individual variances in perceived domain importance or the influence of socio-economic factors. This current scoring approach also fails to account for the relationship between the social determinants of health or individual qualities or personality traits and student flourishing [9].

There is a significant gap in understanding how individual values, intrinsic factors such as grit and coping style, and external factors such as financial security intersect with a student’s ability to flourish through training. The purpose of this study is to explore how demographics and individual values, qualities, and personality traits are associated with perceptions of flourishing among medical (MD), physician assistant (PA), and nurse practitioner (NP) students.

## Methods

### Setting, sample population, and recruitment

Current MD, PA, and NP students from two academic medical centers were recruited to participate in this cross-sectional quantitative study. Both institutions have traditional campus-based MD programs and a combination of online or hybrid and/or campus-based PA and NP programs. MD-PhD students were included. Additionally, while nursing students pursuing either a masters (Master of Science in Nursing) or doctoral degree (Doctor of Nursing Practice) were included, PhD in Nursing Science students at both institutions were excluded as their programs are not clinical in nature. Students across all years in the identified programs were included. A target response of 310 was set by using a 95% confidence interval with a 5% margin of error on a population size of 1820 students [12]. Survey recruitment occurred between August 6 and October 9, 2023, through a combination of emails, flyers, and announcements during live in-person and online sessions.

The study was approved and determined to be Exempt by the Institutional Review Boards at the Medical University of South Carolina (protocol #00129125) and Yale University (protocol #2000035757).

### Survey design and instrument scoring

The survey was delivered through a secure survey platform [REDCap] and began with an informed consent statement. Participants indicating they agreed with the statement were brought to the survey while those that did not agree were brought to the end of the survey. The survey was estimated to take less than 20 minutes. The 79 survey questions included a series of demographic questions including their professional training program and current year in training. Additionally, participants were asked questions about their financial stability and their considerations for leaving training. The following validated instruments were included: Secure Flourish Index (SFI) [8], the WellRx [13, 14], the 2 Question Maslach Burnout Inventory (2QMBI) [15, 16], Brief COPE Inventory (COPE) [17, 18], the Short Grit Scale (S-GRIT) [19], and Duke University Religion Index (DUREL) [20]. The SFI measures flourishing using 12 questions, each with an 11-point Likert scale; greater scores suggesting a greater level of flourishing [9]. The WellRx measures the SDOH using 11 yes/no questions; greater scores suggesting a greater SDOH needs [13, 14]. The instrument was modified to omit one question regarding education access as all participants are currently enrolled in a masters or doctoral level program. One question was added regarding access to mental and physical health care to align with the Health People 2030 definition of the SDOH [13]. The 2QMBI measures burnout using two questions, each with a seven-point Likert scale; a score of greater than three on either item suggests greater burnout [15, 16]. The COPE measures coping approach through 28 questions, each with a four-point Likert scale to produce three coping style subscales: problem-focused, emotion-focused, and avoidant [17, 18]. Greater subscale scores suggest greater use of the coping style. The S-GRIT measures grittiness using 8 questions, each with a five-point Likert scale; greater scores indicate more grit [19]. The DUREL measures religiosity using six questions, each with either a five- or six-point Likert scale to produce three subscales: organizational religiosity, nonorganizational religiosity, and intrinsic religiosity [20]. Greater subscale scores suggest greater religiosity. Included instruments were scored using the established scoring approaches.

In addition, a novel scoring approach was introduced for the Secure Flourish Index. The SFI was developed to explore broad dimensions of human thriving and has been widely used in many large population studies but has had a more limited use in clinical health professions education [9, 21]. The established SFI scoring approach will be referred to as the traditional approach (tSFI) and the novel approach will be referred to as the self-weighted approach (swSFI). In a prior analysis conducted by the research team, the Bland-Altman plot was used to assess the agreement in scores between the tSFI and swSFI scoring approaches (see scoring approach in S1 Table) [22]. It was established that there was disagreement between the two scoring approaches meaning when flourishing domain weights were applied by the participant, the scores were not in agreement with the scores using the traditional approach. This presented a need to further explore how individual values may be related to perceived flourishing and what factors influence how students weight the relative importance of flourishing domains. Because of this, both scoring approaches (tSFI and swSFI) will be used in this analysis.

### Self-weighted Secure Flourish Index scoring

The novel swSFI was employed to create a values-based flourishing score for each participant. Participants were presented with the six domains of flourishing as established in the SFI. Students were asked to, “assign a percentage to each area indicating how important you believe the topic is to your ability to flourish in life. The total percentages assigned should equal 100%.” Topics perceived to be more important to their individual flourishing are reflected by a higher relative percentage. The individual category percentage weights were then applied to the individual SFI responses to produce a novel, self-weighted SFI score for each participant. As there is currently no established cutoff score to indicate flourishing versus non-flourishing using the SFI, results are presented using both the tSFI and the swSFI (S1 Table for scoring example). For cases missing one SFI response, data were imputed to add the score of the other question in the given domain. For cases missing one WellRx response, a score of zero was imputed. Records missing more than one SFI or WellRx response were excluded from the analysis. For all other analyses, participants were only excluded for individual analyses where there were incomplete instrument or demographic responses but included in all analyses involving completed responses. The complete survey can be found in S1 File.

### Analysis

Descriptive statistics were calculated for demographic characteristics. Means and standard deviations for both the tSFI and swSFI were calculated. For both scoring approaches, the total flourishing score (tSFI or swSFI) was used as a continuous variable. Continuous sociodemographic variables were correlated with both the tSFI and swSFI scores, as well as the individual domain scores for the tSFI. For categorical variables, mean tSFI, swSFI, and domain scores were compared across groups. The percentage weights for each domain were also used in the comparison of means and correlation analyses; these results are presented in S2 Table. Two-tailed t-test, analysis of variance (ANOVA), or Pearson’s correlation were used, using non-parametric equivalents when assumption(s) were violated. SPSS version 28 was used for data analysis with significance level set to an alpha of 0.05.

Multiple linear regression was used to assess possible predictors of tSFI and swSFI scores. Independent variables for the regression were purposively selected using the outcomes of the comparison of means and correlation analyses based on a p-value ≤ .2 to minimize the chance of a type II error, as a traditional significance cutoff of p < .05 may fail to capture variables that are not statistically significant but of clinical importance. Collinearity was assessed and no variables were excluded.

## Results

A total of 393 of 1820 eligible students began the survey (21.6%). There were 280 students (15.4%) who completed the SFI, the self-weighted SFI, and the WellRx. The average age was 28.7 years (SD 6.5, n = 269), and the sample was 76.7% female (n = 211/275), 70.4% White or White and another race (n = 197/280), and 23.4% first generation students (n = 64/274; Table 1). There were 14.9% (n = 41/275) of students who reported having seriously considered leaving training in the past six months, with personal mental health being the leading cause consideration (65.9%, n = 27/41) followed by financial stress (56.1%, n = 23/41).

**Table 1:** Demographic and descriptive data N = 280.

	n	(%)	
**Training Profession**	**280**		
Medical Doctor	95	33.9	
Nurse Practitioner	59	21.1	
Physician Assistant/Associate	126	45.0	
**Gender Identity**	275		
Man	61	22.2	
Woman	211	76.7	
Other/Prefer not to disclose	3	1.1	
**Race** ^a^	**280**		
White	197	70.4	
Black or African American	17	6.1	
American Indian or Alaskan Native	5	1.8	
Asian	49	17.5	
Native Hawaiian or Pacific Islander	0	0.0	
Prefer not to answer	17	6.1	
**Hispanic, Latinx, or Spanish**	**274**		
Yes	16	5.8	
**Year in training**	**275**		
<3 months	59	21.5	
3 months– 1 year	67	24.4	
Year 2	91	33.1	
Year 3	32	11.6	
Year 4	26	9.5	
**Relationship status**	**275**		
Single	172	62.5	
Married/Domestic Partner/Civil Union	96	34.9	
Separated/Divorced/Widowed	7	2.6	
**Parental Undergraduate Degree Status**	**274**		
Yes, both parents	158	57.7	
Yes, one parent	52	19.0	
No, neither parent	64	23.4	
**Seriously considered dropping out in the past 6 months?**	**275**		
Yes	41	14.9	
**Factors influencing drop out considerations**	**41**		
Personal mental health	27	65.9	
Personal physical illness	4	9.8	
Family stress	16	39.0	
Financial stress	23	56.1	
Difficulty of course work	10	24.4	
Lack of connection to the program	11	26.8	
Something else	6	14.6	
	**n**	**M(SD)**	**median(Q1:Q3)**
**Percentage Education Expenses from Each** ^b^	**271**		
Student loans		41.1(36.1)	40.0(0.0:75.0)
Personal cash/savings (including spouse)		11.9(20.5)	5.0(0.0:10.0)
Family cash/savings (extended family)		21.8(32.2)	2.0(0.0:32.0)
Scholarships/grants (not including Pell Grant)		19.9(29.4)	5.0(0.0:34.0)
Pell Grant		2.5(8.1)	0.0(0.0:0.0)
GI Bill		1.5(10.1)	0.0(0.0:0.0)
Something else		1.0(8.8)	0.0(0.0:0.0)
**Percentage Living Expenses from Each** ^b^	**272**		
Student loans		25.5(36.1)	0.0(0.0:50.0)
Personal cash/savings (including spouse)		44.3(41.5)	30.0(0.3:95.0)
Family cash/savings (extended family)		16.2(30.2)	0.0(0.0:18.8)
Scholarships/grants		10.6(27.8)	0.0(0.0:0.0)
Federal/state assistance		0.44(3.1)	0.0(0.0:0.0)
Something else		2.7(14.3)	0.0(0.0:0.0)
**Age, years**	**269**	28.7(6.5)	26.0(24.0:32.0)
**Number of legal dependents**	**267**	0.5(1.0)	0.0(0.0:0.0)
**Brief COPE Inventory**			
Avoidant^c^	264	1.6(0.4)	1.5(1.4:1.8)
Emotion^c^	259	2.3(0.5)	2.3(2.0:2.7)
Problem^c^	260	2.6(0.6)	2.8(2.3:3.1)
**Short-GRIT** ^c^	**257**	3.5(0.6)	3.6(3.1:4.0)
**2QMBI** ^c^ ^,^ ^d^	**275**	5.3(3.2)	5.0(2.0:8.0)
**DUREL**			
Organized^c^	**262**	2.4(1.6)	2.0(1.0:4.0)
Nonorganized^c^	**262**	2.3(1.7)	1.0(1.0:4.0)
Intrinsic^c^	**258**	8.5(4.6)	8.0(3.0:13.0)
**WellRx** ^c^ ^,^ ^e^	**280**	1.1(1.3)	1.0(0.0:2.0)

Abbreviations: 2QMBI: 2 question Maslach Burnout Inventory; DUREL: Duke University Religion Index

^a^Race was asked in a ‘select all that apply’ format so total percentage equates to more than 100%

^b^Cases with total expense percentages > or <100% were not included in the demographics reporting

^c^Instrument name (possible score range): COPE Avoidant (1–4); COPE Emotion (1–4); COPE Problem (1–4); GRIT (1–5); 2QMBI (0–12); DUREL Organizational Religiosity (1–6); DUREL Nonorganizational Religiosity (1–5); DUREL Intrinsic Religiosity (3–15); WellRx (0–11)

^d^The two questions in the 2QMBI were adjusted to fit the sample population by changing the words “job” and “work” to “training.”

^e^One question was added to the WellRx asking about access to physical and mental health care to meet the Social Determinants of Health domain of health care access and quality. One question on access to education was omitted as all students are currently enrolled in a graduate or doctoral program

### Weighting of flourishing domains

**Fig 1:**
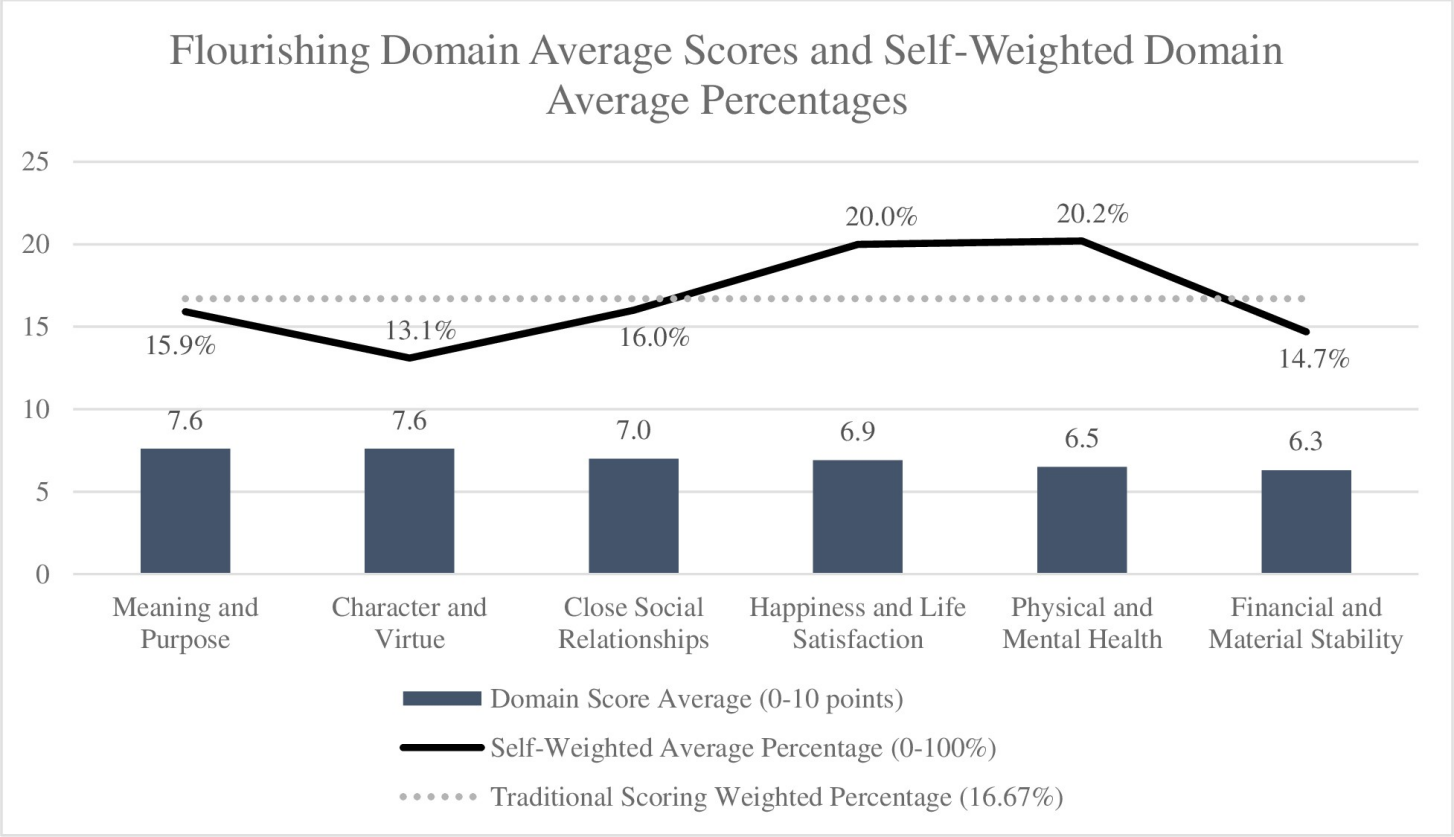
Flourishing domain average scores and self-weighted domain average percentages.

There is currently no established threshold score to indicate flourishing using the SFI, but higher scores indicate greater flourishing. The swSFI produced an almost equivalent mean flourishing score as the tSFI (M 83.9, SD 16.3 and M 84.0, SD 15.0, respectively; Table 2). If all domains were to be weighted equally as done in the traditional SFI scoring, each domain score would account for 16.67% of total flourishing. However, when students were asked to weight these domains themselves, the domain of physical and mental health was weighted with the highest relative importance with a mean of 20.2% (SD 8.4), followed by happiness and life satisfaction at 20.0% (SD 9.0). While the domains of meaning and purpose and character and virtue had the highest mean domain scores (M 7.62, SD 1.7 and M 7.60, SD 1.3, respectively), character and virtue was weighted with the lowest relative percentage domain weight in the swSFI (M 13.1% (SD 6.5)) indicating that while students have relatively high levels of flourishing in the domain of character and virtue, they do not perceive this domain to be as important to their individual overall flourishing (Table 2**)**. Conversely, physical and mental health had the highest relative percentage weight, but was second to lowest in mean domain score (mean 6.5, SD 1.7); while individuals place high value on this domain, they report relatively low flourishing (Table 2). Participants had the lowest mean domain score in financial and material stability (M 6.3, SD 2.7) and weighted this domain with the second lowest overall percentage (M 14.7%, SD 8.6). Average domain scores and average domain percentage weights versus the traditional equal weights are compared in **Fig 1**.

**Table 2:** Traditional Secure Flourish Index total and domain scores and self-weighted secure flourish index total and domain percentage weights.

	n	M(SD)	median(Q3-Q1)
**Traditional Secure Flourish Index**	**280**	84.0(15.0)	86.0(73.3:95.0)
** Flourishing Domains**	**Domain rank**	**Domain Score**	
		**Possible Range 0–10**	
Meaning and Purpose	1	7.62(1.7)	8.0(6.5:9.0)
Character and Virtue	2	7.60(1.3)	7.5(5.6:8.5)
Close Social Relationships	3	7.0(2.1)	7.5(5.6:8.5)
Happiness and Life Satisfaction	4	6.9(1.6)	7.0(6.0:8.0)
Physical and Mental Health	5	6.5(1.7)	7.0(5.5:8.0)
Financial and Material Stability	6	6.3(2.7)	6.5(4.5:8.9)
**Self-weighted Secure Flourishing Index**	**280**	83.9(16.3)	86.7(73.8:96.3)
** Flourishing Domains**	**Domain rank**	**Domain Weight**	
		**Possible Range 0–100%**	
Physical and Mental Health	1	20.2(8.4)	20.0(15.0:20.0)
Happiness and Life Satisfaction	2	20.0(9.0)	20.0(15.0:20.0)
Close Social Relationships	3	16.0(7.5)	15.0(10.0:20.0)
Meaning and Purpose	4	15.9(8.3)	15.0(20.0:20.0)
Financial and Material Stability	5	14.7(8.6)	14.0(10.0:20.0)
Character and Virtue	6	13.1(6.5)	12.0(10.0:16.0)

### Correlation

For the tSFI, total flourishing scores were higher with higher grit (r = .37, p<0.001). Conversely, tSFI scores were lower with higher social needs (WellRx; r = -.34, p<0.001), burnout (r = -.47, p<0.001), or avoidant coping style (r = -.45, p<0.001; Table 3). Similarly, for the swSFI, flourishing scores were higher with higher grit (r = .35, p<0.001) and lower with higher social needs (WellRx; r = -.33, p<0.001), burnout (r = -.46, p<0.001), or avoidant coping styles (r = -.44, p<0.001; Table 3). Additionally, there is a moderate negative correlation between avoidant coping and the tSFI domain specific scores for happiness and life satisfaction (r = -.40, p<0.001), physical and mental health (r = -.43, p<0.001), and meaning and purpose (r = -.38, p<0.001). The strongest positive correlations were found between grit and the domain scores of meaning and purpose (r = .34, p<0.001) and character and virtue (r = .32, p<0.001; Table 4).

**Table 3:** Comparison of means and correlation testing for independent variables using the traditional Secure Flourish Index and the self-weighted secure flourish index total score (N = 280).

		Total tSFI Score		Total swSFI Score	
Category	n	Mean(SD)	p-value	Mean(SD)	p-value
**Training Profession**	**280**	Possible		Possible	
		Range 0–120		Range 0–120	
Medical Doctor	95	83.0 (13.1)	.54^+^	83.5 (14.1)	.60^+^
Physician Assistant	126	84.8 (15.4)		84.8 (16.6)	
Nurse Practitioner	59	83.9 (17.2)		82.9 (19.0)	
**Stage in training**	**275**				
<3 months	59	85.0 (12.9)	.04^+^^a^	85.4 (15.4)	.02^+^^a^
3 months– 1 year	67	87.7 (15.1)		87.8 (16.0)	
Year 2	91	82.5 (14.9)		82.2 (15.8)	
Year 3 or 4	58	81.3 (16.9)		81.0 (18.1)	
**Marital Status**	**275**				
Married, Domestic Partner, or Civil Union	96	86.7 (15.8)	.04	86.4 (17.1)	.05^+^
Single, Separated, Divorced, or Widowed	179	82.6 (14.6)		82.7 (15.9)	
**Race** ^b^	**257**				
White	187	84.4 (15.6)	.48	84.2 (16.8)	.65^+^
BIPOC	70	82.9 (14.2)		83.1 (15.8)	
**Gender identity** ^c^	**272**				
Man	61	86.3 (14.8)	.21	86.7 (15.3)	.20^+^
Woman	211	83.6 (15.2)		83.5 (16.5)	
**First generation status** ^d^	**274**				
Yes	64	84.0 (15.7)	.98	83.6 (16.5)	.77^+^
No	210	84.1 (15.0)		84.1 (16.4)	
**Considered dropping out** ^e^	**275**				
Yes	41	75.3 (16.2)	< .001	74.4 (18.3)	< .001^+^
No	234	85.6 (14.4)		85.7 (15.5)	
		**Total tSFI Score**		**Total swSFI Score**	
	**n**	**r**	**p-value**	**r**	**p-value**
**Age**	269	.04	.53^+^	.02	.79^+^
**Number of Dependents**	267	.14	.03^+^	.13	.04^+^
**WellRx** ^f^	280	-.34	< .001^+^	-.33	< .001^+^
**COPE**					
Avoidant	264	-.45	< .001	-.44	< .001^+^
Emotion	259	-.13	.04	-.13	.04^+^
Problem	260	.07	.24	.09	.13^+^
**Short-GRIT**	257	.37	< .001	.35	< .001^+^
**2QMBI** ^g^	275	-.47	< .001	-.46	< .001^+^
**DUREL**					
Organized Religion	262	.15	.01^+^	.14	.03^+^
Non-organized	262	.12	.05^+^	.10	.10^+^
Religion					
Intrinsic Spirituality	258	.19	< .01	.17	< .01^+^
**Education Expenses** ^h^	271	.08	.20	.03	.57^+^
**Living Expenses** ^h^	272	-.03	.58^+^	-.04	.50^+^

Abbreviations: tSFI: traditional Secure Flourish Index; swSFI: self-weighted Secure Flourish Index; BIPOC: Black, Indigenous, and People of Color; 2QMBI: 2 question Maslach Burnout Inventory; DUREL: Duke University Religion Index

^+^Indicates that nonparametric testing used (Kruskal-Wallis, Mann-Whitney U, or Spearman Correlation)

^a^Post-hoc testing with Bonferroni correction resulted in no significant difference between group means.

^b^Race “white” includes participants who selected only “white” and “BIPOC” includes any participant who selected White and another race or only another race(s). Race was asked in a ‘select all that apply’ format so total percentage equates to more than 100%.

^c^For analysis of gender, cases were recoded into male and female; participants selecting ‘other’ or ‘prefer to self-describe’ were recoded as missing.

^d^First generation status “yes” includes participants who have no parent(s) with a college degree.

^e^Includes participants who answered “yes” to the question “have you seriously considered dropping out of training the past 6 months.”

^f^ One question was added to the WellRx asking about access to physical and mental health care to meet the Social Determinants of Health domain of health care access and quality. One question on access to education was omitted as all students are currently enrolled in a graduate or doctoral program.

^g^The two questions in the 2QMBI were adjusted to fit the sample population by changing the words “job” and “work” to “training.”

^h^Represents the percentage of total each education and living expenses paid for with student loans. Cases with total expense percentages > or <100% were not included.

**Table 4:** Comparison of means and correlation testing for independent variables using the traditional Secure Flourish Index domain scores.

		Traditional Secure Flourish Index (tSFI) Domain Scores											
		Happiness and Life Satisfaction		Physical and Mental Health		Meaning and Purpose		Character and Virtue		Close Social Relationships		Financial and Material Stability	
		Possible range 0–10											
Category	n	Mean(SD)		Mean(SD)		Mean(SD)		Mean(SD)		Mean(SD)		Mean(SD)	
**Training Profession**	**280**												
Medical Doctor	95	7.0(1.3)		6.6(1.6)		7.4(1.6)		7.1(1.4)		6.6(1.9)		6.7(2.6)	
Physician Assistant	126	6.9(1.8)		6.6(1.8)		7.8(1.7)		7.8(1.3)		7.3(2.2)		5.9(2.8)	
Nurse Practitioner	59	6.6(1.8)		6.2(1.7)		7.6(1.9)		8.0(1.2)		6.8(2.2)		6.7(2.7)	
p-value		.51^+^		.17^+^		.06^+^		< .001		< .01^+^		.06^+^	
**Stage in training**	**275**												
<3 months	59	7.2(1.5)		6.7(1.6)		7.6(1.6)		7.3(1.2)		7.0(1.7)		6.6(2.7)	
3 months– 1 year	67	7.1(1.8)		6.8(1.8)		8.2(1.6)		7.8(1.3)		7.6(2.0)		6.3(2.6)	
Year 2	91	6.7(1.7)		6.4(1.7)		7.5(1.8)		7.7(1.3)		7.0(2.1)		6.0(2.8)	
Year 3 or 4	58	6.7(1.6)		6.2(1.7)		7.3(1.9)		7.5(1.5)		6.3(2.4)		6.7(2.7)	
p-value		.03^+^		.12		.01^+^		.16		< .01^+^		.38^+^	
**Marital Status**	**275**												
Married, Domestic Partner, or Civil Union	96	7.0(1.7)		6.7(1.7)		8.0(1.6)		7.8(1.4)		7.2(2.2)		6.7(2.6)	
Single, Separated, Divorced, or Widowed	179	6.9(1.6)		6.5(1.7)		7.4(1.8)		7.5(1.3)		7.0(2.1)		6.7(2.8)	
p-value		.39		.30		< .01^+^		.11		.27		.08	
**Race** ^a^	**257**												
White	187	6.9(1.7)		6.6(1.7)		7.7(1.8)		7.7(1.4)		7.2(2.1)		6.2(2.8)	
BIPOC	70	6.9(1.6)		6.3(1.7)		7.5(1.8)		7.5(1.2)		6.6(2.3)		6.7(2.5)	
p-value		.82		.22		.41^+^		.39		.12^+^		.28^+^	
**Gender identity** ^b^	**272**												
Man	61	7.1(1.6)		6.8(1.5)		7.9(1.6)		7.6(1.1)		7.1(2.0)		6.7(2.9)	
Woman	211	6.8(1.7)		6.5(1.7)		7.6(1.8)		7.6(1.4)		7.0(2.1)		6.2(2.7)	
p-value		.22^+^		.16		.36^+^		.62^+^		.79^+^		.20^+^	
**First generation status** ^c^	**274**												
Yes	64	6.8(1.9)		6.5(1.8)		7.7(1.8)		8.0(1.1)		6.8(2.3)		6.2(2.5)	
No	210	6.9(1.6)		6.6(1.7)		7.6(1.7)		7.5(1.4)		7.1(2.1)		6.3(2.8)	
p-value		.49		.71		.62^+^		.01		.39^+^		.64^+^	
**Considered dropping out** ^d^	**275**												
Yes	41	5.8(1.9)		5.7(1.8)		6.8(2.0)		7.4(1.4)		6.4(2.3)		5.6(2.9)	
No	234	7.1(1.5)		6.7(1.7)		7.8(1.7)		7.6(1.3)		7.1(2.0)		6.5(2.7)	
p-value		< .001^+^		< .001		< .01^+^		.21		.07^+^		.06^+^	
		**Traditional Secure Flourish Index (tSFI) Domain Scores**											
		**Happiness and Life Satisfaction**		**Physical and Mental Health**		**Meaning and Purpose**		**Character and Virtue**		**Close Social Relationships**		**Financial and Material Stability**	
	**n**	**r**	**p-value**	**r**	**p-value**	**r**	**p-value**	**r**	**p-value**	**r**	**p-value**	**r**	**p-value**
**Age**	269	-.02	.71	.03	.58	.14	.02	.19	< .01	.03	.60	.06	.32
**Dependents**	269	.05	.43^+^	.04	.57^+^	.25	< .001^+^	.16	< .01^+^	.11	.09^+^	.07	.24^+^
**WellRx** ^e^	280	-.21	< .001	-.26	< .001	-.15	.02	-.09	.15	-.28	< .001	-.34	< .001
**COPE**													
Avoidant	264	-.40	< .001	-.43	< .001	-.38	< .001	-.24	< .001	-.32	< .001	-.15	.01
Emotion	259	-.17	< .01	-.25	< .001	-.50	.42	.09	.14	-.06	.33	-.06	.31
Problem	260	.01	.85	.62	.16	.09	< .001	.25	.35	.06	.43	-.05	.87
**Short-GRIT**	257	.22	< .001	.24	< .001	.34	< .001	.32	< .001	.23	< .001	.19	< .01
**2QMBI** ^f^	275	-.47	< .001	-.47	< .001	-.34	< .001	-.11	.06	-.29	< .001	-.22	< .001
**DUREL**													
Organized Religion	262	.08	.22	.08	.21	.21	< .001	.19	< .01	.10	.11	.02	.72
Non-organized Religion	262	.06	.33	.02	.71	.23	< .001	.20	< .01	.06	.30	.04	.57
Intrinsic Spirituality	258	.07	.23	.02	.74	.25	< .001	.29	< .001	.18	< .01	.03	.66
**Education Expense** ^g^	271	-.03	.59	.01	.92	.12	.06	.21	< .001	.18	< .01	-.08	.20
**Living Expenses** ^g^	272	-.02	.78^+^	.03	.58^+^	.05	.46^+^	.10	.01^+^	.12	.06^+^	-.21	< .001^+^

^+^Indicates that nonparametric testing used (Kruskal-Wallis, Mann-Whitney U, or Spearman Correlation)

Abbreviations: tSFI: traditional Secure Flourish Index; BIPOC: Black, Indigenous, and People of Color; 2QMBI: 2 question Maslach Burnout Inventory; DUREL: Duke University Religion Index

^a^Race “white” includes participants who selected only “white” and “BIPOC” includes any participant who selected White and another race or only another race(s). Race was asked in a ‘select all that apply’ format so total percentage equates to more than 100%.

^b^For analysis of gender, cases were recoded into male and female; participants selecting ‘other’ or ‘prefer to self-describe’ were recoded as missing.

^c^First generation status “yes” includes participants who have no parent(s) with a college degree.

^d^Includes participants who answered “yes” to the question “have you seriously considered dropping out of training the past 6 months.”

^e^ One question was added to the WellRx asking about access to physical and mental health care to meet the Social Determinants of Health domain of health care access and quality. One question on access to education was omitted as all students are currently enrolled in a graduate or doctoral program.

^f^The two questions in the 2QMBI were adjusted to fit the sample population by changing the words “job” and “work” to “training.”

^g^Represents the percentage of total each education and living expenses paid for with student loans. Cases with total expense percentages > or <100% were not included.

### Comparison of means

Traditional SFI scores were statistically significantly lower (p<0.001) for students who had considered leaving training in the past 6 months compared to those who had not (M 75.3, SD 16.2 vs. M 85.6, SD 14.4, respectively). Student swSFI flourishing scores were also statistically significantly lower (p<0.001) for students who had considered leaving training in the past 6 months (M 74.4, SD 18.3) than those who had not (M 85.7, SD 15.5; p<0.001). For current stage of training, Kruskal-Wallis testing resulted in a statistically significant difference for both tSFI (p = 0.04) and swSFI (p = 0.02) but post-hoc testing with Bonferroni correction for multiple groups resulted in no statistically significant differences between groups. However, mean flourishing for both the total tSFI and swSFI are highest in the 3 months to 1 year group (tSFI M 87.7, SD 15.1; swSFI M 87.8, SD 16.0) and lowest in years 3 and 4 group (tSFI M 81.3, SD 16.9; swSFI M 81.0, SD 18.1). There is also a 1.3 point difference in the mean domain score for close social relationships between students in the 3 months to 1 year group (M 7.6, SD 2.0) to those in years 3 or 4 group (M 6.3, SD 2.4; p<0.01). Students who considered leaving their training in the past 6 months had statistically significantly lower domain scores than those who had not for every domain except character and virtue and close social relationships. Flourishing scores did not significantly differ between the two institutions for the tSFI (M 84.1, SD 14.3 vs M 83.9, SD 15.9; p = 0.90) or the swSFI (M 84.4, SD 15.7 vs 83.4 SD 17.0, p = 0.59) despite differing enrollment across professional groups, therefore only training profession was included in the main analysis. Tables 3 and 4 include complete results for comparison of means. S2 Table contains correlation and comparison of means for weighted domain percentages.

### Regression analysis

Regression results (Table 5) indicate that the overall model including all potential predictors simultaneously significantly predicts traditional Secure Flourish Index score [R^2^ = .53, R^2^_adj_ = .50, F(15, 221) = 16.8, p < .001]. The tSFI regression model is tSFI = 80.0–14.4(COPE avoidant score) + 5.1(Dropout Not Considered: Yes/No) + 4.1(GRIT score)– 2.7(WellRx Score)– 1.7(2QMBI score) + 0.1(%Education paid by student loans).

**Table 5:** Multivariable linear regression association with individual total Secure Flourish Index scores with traditional and self-weighted scoring approaches.

	B	SE	95% CI	t-statistic	p-value
**Traditional Secure Flourish Index (tSFI)**					
Number legal dependents	1.3	.9	-.4, 3.0	1.5	.13
WellRx^a^	-2.7	.6	-4.0, -1.5	-4.3	< .001
COPE Avoidant	-14.4	2.4	-19.0, -9.7	-6.1	< .001
COPE Emotion	1.5	1.7	-1.8, 4.9	.9	.37
S-Grit	4.1	1.3	1.4, 6.7	3.0	< .01
2QMBI^b^	-1.7	.3	-2.2, -1.2	-6.4	< .001
DUREL organizational	.5	.7	-.9, 1.9	.7	.50
DUREL nonorganizational	-1.2	.6	-2.4, .1	-1.9	.06
DUREL intrinsic	.4	.3	-.2, .9	1.4	.16
% Education Loans^c^	.1	.02	.01, .1	2.6	.01
Drop out consideration^d^					
Yes (Ref)	-	-	-	-	-
No	5.1	2.3	.6, 9.5	2.2	.03
Marital Status^e^					
Not Married (Ref)	-	-	-	-	-
Married	2.8	1.8	-.7, 6.3	1.6	.12
Training Stage					
<3mo (Ref)	-	-	-	-	-
3mo-1 year	3.6	2.4	-1.1, 8.3	1.5	.13
Year 2	3.8	2.2	-.6, 8.2	1.7	.09
Years 3–4	2.9	2.3	-1.8, 7.5	1.2	.22
**Self-weighted Secure Flourish Index (swSFI)**					
	**B**	**SE**	**95% CI**	**t-statistic**	**p-value**
Number legal dependents	1.5	1.0	-.5, 3.4	1.5	.14
WellRx^a^	-2.3	.7	-3.8, -.9	-3.3	< .01
COPE Avoidant	-14.6	2.7	-19.9, -9.3	-5.4	< .001
COPE Emotion	-1.7	2.7	-7.1, 3.7	-.6	.53
COPE Problem	3.5	1.9	-.4, 7.3	1.8	.08
S-Grit	3.3	1.5	.3, 6.4	2.1	.03
2QMBI^b^	-1.6	.3	-2.3, -1.0	-5.3	< .001
DUREL organizational	.7	.8	-.9, 2.2	.8	.42
DUREL nonorganizational	-1.5	.7	-3.0, -.1	-2.1	.03
DUREL intrinsic	.5	.3	-.1, 1.1	1.7	.09
Drop out consideration^d^					
Yes (Ref)	-	-	-	-	-
No	5.5	2.6	.4, 10.7	2.1	.03
Marital Status^e^					
Not Married (Ref)	-	-	-	-	-
Married	3.5	2.0	-.5, 7.5	1.7	.09
Training Stage					
<3mo (Ref)	-	-	-	-	-
3mo-1 year	3.9	2.6	-1.3, 9.0	1.5	.14
Year 2	3.2	2.5	-1.7, 8.2	1.3	.20
Years 3–4	1.8	2.7	-3.4, 7.1	.8	.50
Gender Identity^f^					
Male (Ref)	-	-	-	-	-
Female	.7	2.1	-3.4, 4.8	.3	.75

Abbreviations: 2QMBI: 2 question Maslach Burnout Inventory; DUREL: Duke University Religion Index

^a^One question was added to the WellRx asking about access to physical and mental health care to meet the Social Determinants of Health domain of health care access and quality. One question on access to education was omitted as all students are currently enrolled in a graduate or doctoral program.

^b^The two questions in the 2QMBI were adjusted to fit the sample population by changing the words “job” and “work” to “training.”

^c^Represents the percentage of total each education and living expenses paid for with student loans. Cases with total expense percentages > or <100% were not included.

^d^Includes participants who answered “yes” to the question “have you seriously considered dropping out of training the past 6 months.”

^e^For analysis of marital status, responses of Married, Domestic Partner, Civil Union were recoded to ‘married’ while Single, Separated, Divorced, Widowed were recoded to ‘not married.’

^f^For analysis of gender, cases were recoded into male and female; participants selecting ‘other’ or ‘prefer to self-describe’ were recoded as missing.

Similarly, regression results indicate that the overall model significantly predicts self-weighted Secure Flourish Index score [R^2^ = .48, R^2^_adj_ = .44, F(16, 215) = 12.4, p < .001]. The swSFI regression model is swSFI = 79.3–14.6(COPE avoidant score) + 5.5(Dropout not considered: Yes/No) + 3.3(GRIT score) - 2.3(WellRx Score) - 1.6(2QMBI score) - 1.5(DUREL_nonorganizational).

## Discussion

This study explores how MD, PA, and NP students value the SFI domains in their overall ability to flourish. The mean score of the tSFI of 84 (SD 15.0), of a possible score of 120, suggests that these students are flourishing overall, as these scores were similar to existing data for attending physicians [23, 24]. Similar data for practicing NPs and PAs does not currently exist. However, there are no validated cut-offs to define flourishing.

### Individual values

Students weighted mental and physical health as the most important domain in their ability to flourish in life. However, mental and physical health had the second lowest domain-specific flourishing score highlighting a large discrepancy in what students value and their current sense of flourishing. Equally weighting the six flourishing domains as done in the traditional SFI fails to account for these differences in individual values or the potential overlap of these domains as they contribute to flourishing. However, as the study of flourishing shifts to explore possible interventions, targeting the mental and physical health domain, which is perceived to be lacking but highly valued by students, may provide a strong starting point. Expanding access to mental health resources and opportunities to engage in physical activity for students may drive improvements in overall flourishing. Additionally, consideration is needed for the potential discordance between mental health and physical health, as students may face unique challenges in one area that are not present in another.

Conversely, students had high relative domain scores in character and virtue but perceived this domain to be the least important to their ability to flourish. Character and virtue encompass the ability put other’s needs above one’s own and to delay personal happiness now for future happiness. High flourishing in this domain aligns with both the duration of medical and nursing training and the servant nature of the professions. However, the low relative self-weighting of this domain emphasizes that while foundational aspects of training, students perceive their individual flourishing to be more heavily influenced by other domains, such as happiness and life satisfaction. Happiness and life satisfaction are more in-the-moment views of wellness in contrast to the delayed gratification associated with the virtue domain. Although highly valued among students, their domain specific happiness and life satisfaction scores are relatively low. While students value this hedonic happiness, similar to their health, they are not currently feeling as fulfilled in this aspect of life.

### Coping and grit

Intrinsic and extrinsic factors are intertwined with students’ perceived flourishing throughout training. Students’ ability to cope with and persevere through hardships are correlated with flourishing. The least commonly used coping style, avoidant coping, significantly predicted flourishing for both the traditional and self-weighted flourishing scores in regression modeling; higher use of avoidant coping resulted in significantly lower flourishing scores. Additionally, both avoidant and emotion driven coping were negatively correlated with tSFI and swSFI scores, while grit was positively correlated. This provides an opportunity for future interventions focused on teaching foundational coping skills to students to reduce avoidant coping as an approach to promote flourishing and possibly mitigate the mental health challenges facing health care providers today.

### Spirituality and flourishing

While there are limited existing data on the perceived importance of flourishing domains, a 2021 general population study found similar results as participants heavily valued physical health and emotional well-being and placed less value on character [10]. However, there are also discrepancies with our findings, as this same study found that daily spiritual practice was a strong predictor for domain importance in every domain but financial security [10]. A recent systematic review found that spirituality is connected to better health outcomes and patient care, recommending that spiritual care education be integrated into medical education [25]. Participation in religious activities was not found to be a strong correlate of overall flourishing in either the tSFI or swSFI models. However, the statistically significant yet weak (r < 0.3) correlation between the DUREL subscales and multiple flourishing domains highlights an important area for future exploration to promote flourishing among students as participation in religious activities can also serve to promote other domains such as social relationships. Religious observance and spirituality have previously been strongly correlated with well-being. The lack of correlation in this cohort may reflect a generational shift away from religion and spirituality [26].

### The social determinants of health

Prior literature shows that clinical health professions students view social relationships as a key component of mental health, which is supported by the relatively high weight given to this domain [27–29]. Students with higher tSFI and swSFI scores were also found to have lower burnout and burnout is negatively correlated with flourishing, which is consistent with prior studies in flourishing among medical students [21]. Additionally, financial stability is highly valued among the general population, whereas our results found financial and material stability to not only have a low domain specific score, but also a low relative value [10]. Many students face financial difficulties throughout their training and these findings may be explained by the concept of adaptive preference, where individuals begin to devalue things that are perceived to be less attainable.

Higher SDOH needs are negatively correlated with flourishing. While this includes factors such as economic need, it also includes the social and community context in which students live and learn as well as their access to health care, the most highly weighted domain. Students with higher social and economic needs are disproportionately negatively affected by challenges during training which often results in increased psychological and financial burden and decreased graduation rates [30, 31]. Improving support systems for students with unmet needs is possibly an integral step in increasing access to and retention of students through medical and nursing training.

While there was no significant difference found in mean flourishing scores between professional training programs, there was a clinically relevant difference found between students in different stages of training, with those in years 3 and 4 having over 6 points lower tSFI scores than those between 3 months and 1 year of training. Further exploration into the role of degree progression and how this may overlap with social and economic needs in the context of flourishing is needed. Additionally, the affiliation of the researchers with the included programs could have dissuaded students from participating or influenced the responses provided.

### Strengths and limitations

This is the first study to our knowledge that weights the domains of the SFI to help faculty and administrators understand the flourishing priorities of our trainees. This is a novel, innovative adaptation of a previously validated instrument that provides insight into the unique experiences of students in a way that may help inform more individualized forms of student support. This study includes PA and graduate-level nursing students, in addition to medical students, whereas prior studies of flourishing are usually limited to only one student type or are focused on non-student populations [21, 32]. The response rate was low, but similar to a response rate for similar studies [10, 21]. However, the low response rate provides a potentially biased sample and reduces the generalizability of the findings. While there was a broad range of flourishing scores, this may have captured students who have a stronger interest in mental health, either positive or negative, resulting in more responses on the ends of the flourishing scale rather than a true cross section of scores. The cross-sectional study design does not allow for understanding of how flourishing changes over time or for identifying causal relationships. The exploration of an existing instrument limits the study of flourishing to established domains and the weighting system applied considers these domains in silos, rather than accounting for potential overlap between the domains in flourishing. How these domains intersect and influence each other is an important area to explore and may best be done through qualitative analysis.

Additionally, the structure did not account for potential differences between online and in-person training or the reasons that an individual would choose one pedagogy over another. The exploration of race dichotomized as White and BIPOC potentially misses the important nuance of experiences and individual stories that are likely shaped by culture and society influence between different groups of individuals. Given the overarching topic of wellness, there is the possibility of response bias. There is also the chance that the study participants are not representative of the larger student population.

### Recommendations for future directions

Further exploration into how individual life experiences influence flourishing across the established domains is needed. Understanding what services are currently being offered by universities to support the individual social and economic needs of students, their utilization, and their effectiveness is an important next step in expanding support. The intersection of flourishing and student achievement, attrition, and SDOH is an important area for continued research. Additionally, implementation and evaluation of activities and support structured on individual values, mitigating avoidant coping, and promotion grit to drive flourishing may help improve overall student wellbeing and reduce attrition rates.

### Conclusions

Students’ social and economic needs are intertwined with their ability to flourish during training. Similarly, avoidant coping through hardships is strongly correlated with decreased flourishing. While students score the flourishing domain of physical and mental health relatively low, they perceive this domain to be the most influential in their ability to flourish. Improving access to mental and physical health care and teaching positive coping through training may be important steps in improving student flourishing. To create supportive environments, we must have a foundational understanding of how life experiences, individual values, and current peer and community support, along with university structure and policy, shape a student’s mental health [33]. Poor mental health significantly impacts student success and is a leading cause of attrition, while improved wellness and flourishing have been shown to decrease thoughts of leaving training [33, 34]. Tailoring support to individual values, reducing avoidant coping, and addressing SDOH needs are important steps in promoting flourishing and improving retention.

## Supporting information

S1 Table Novel self-weighted secure flourish index scoring example. This table contains a scoring example for the calculation of the swSFI score using the tSFI score and the domain weights applied by the participants.
(DOCX)

S2 Table Novel self-weighted Secure Flourish Index domain weighting comparison of means and correlation. This table contains the comparison of means and correlation data for the swSFI six individual SFI domains.
(DOCX)

S1 File Complete survey questions. This file contains the complete survey used in this study.
(PDF)
