# Supplementary material for: Individual values, the social determinants of health, and flourishing among medical, physician assistant, and nurse practitioner students
Source: PLoS One. 2024 Sep 27;19(9):e0308884. doi: 10.1371/journal.pone.0308884 (PMC11432832; doi:10.1371/journal.pone.0308884)
Supplement: S1 Table — This table contains a scoring example for the calculation of the swSFI score using the tSFI score and the domain weights applied by the participants. (DOCX) [file pone.0308884.s001.docx]

**Supplemental 1 Table: Example of Self-Weighted Secure Flourish Index (swSFI) Scoring as Compared to Traditional Scoring (tSFI)**

Participants completed the Secure Flourish Index (SFI) and then were asked to apply a relative percentage weight (0-100%) to each of the established six flourishing domains based on their perceived relative importance to their individual flourishing. These domain weights (percentages) were then applied to the SFI responses to produce a novel, self-weighted SFI score. The established scoring approach will be referred to as the traditional approach (tSFI) and the novel approach will be referred to as the self-weighted approach (swSFI). An example of the comparative scoring approaches is outlined below.

| **Domain** | **Question 1 score** | **Question 2 score** | **Domain average score** | **Weighted %** | **swSFI**  **domain score^a^** | **tSFI domain score^b^** |
| --- | --- | --- | --- | --- | --- | --- |
| Happiness and Life Satisfaction | 8 | 7 | 7.5 | 10 | .75 | 1.25 |
| Mental and Physical Health | 7 | 7 | 7 | 10 | .70 | 1.17 |
| Meaning and Purpose | 6 | 10 | 8 | 45 | 3.60 | 1.33 |
| Character and Virtue | 10 | 10 | 10 | 15 | 1.50 | 1.67 |
| Close Social Relationships | 6 | 5 | 5.5 | 5 | .28 | 0.92 |
| Financial and Material Stability | 9 | 9 | 9 | 15 | 1.35 | 1.50 |
| ***Total score out of 10*** | | | | | **8.18** | **7.84** |
| ***Conversion*** | | | | | **8.18/10 = x/120** | **7.84/10 = x/120** |
| ***Total score out of 120*** | | | | | **98.2** | **94.1** |

^a^Self-weighted domain sum score is calculated by multiplying the average domain score by the weighted % value assigned by the participant

^b^Traditional domain weight sum score is calculated by multiplying the average domain score by 16.667% (calculated by dividing 100% evenly across the six domains)
