# Supplementary material for: Individual values, the social determinants of health, and flourishing among medical, physician assistant, and nurse practitioner students
Source: PLoS One. 2024 Sep 27;19(9):e0308884. doi: 10.1371/journal.pone.0308884 (PMC11432832; doi:10.1371/journal.pone.0308884)
Supplement: S2 Table — This table contains the comparison of means and correlation data for the swSFI six individual SFI domains. (DOCX) [file pone.0308884.s002.docx]

**Supplemental 2 table: Novel Self-weighted Secure Flourish Index Domain Weighting Comparison of Means and Correlation**

**Table 1:** **T-test and one-way ANOVA testing for categorical variables using the self-weighted Secure Flourish Index domain percentage weights (N=280)**

|  |  | **Happiness and Life Satisfaction** | **Physical and Mental Health** | **Meaning and Purpose** | **Character and Virtue** | **Close Social Relationships** | **Financial and Material Stability** |
| --- | --- | --- | --- | --- | --- | --- | --- |
| **Category** | **n** | **Mean%(SD)** | **Mean%(SD)** | **Mean%(SD)** | **Mean%(SD)** | **Mean%(SD)** | **Mean%(SD)** |
| **Training Profession** | **280** |  | | | | | |
| *Medical Doctor* | 95 | 18.4 (7.8) | 20.6 (10.8) | 16.7 (9.8) | 12.8 (6.5) | 16.8 (6.7) | 14.8 (9.7) |
| *Physician Assistant* | 126 | 20.2 (9.7) | 19.8 (6.6) | 15.8 (7.8) | 12.9 (6.5) | 17.0 (8.0) | 14.4 (7.8) |
| *Nurse Practitioner* | 59 | 22.2 (9.0) | 20.5 (7.7) | 15.0 (6.6) | 14.3 (6.7) | 12.7 (6.6) | 15.3 (8.7) |
| *p-value* |  | .03^+^ | .76^+^ | .44 | .35^+^ | <.001 | .78 |
| **Stage in training** | **275** |  | | | | | |
| *<3 months* | 59 | 19.9 (11.1) | 20.4 (11.7) | 16.9 (7.9) | 13.0 (7.0) | 16.5 (6.5) | 13.4 (7.0) |
| *3 months – 1 year* | 67 | 20.3 (9.0) | 19.3 (6.8) | 17.0 (7.7) | 12.7 (5.7) | 16.3 (8.5) | 14.5 (8.3) |
| *Year 2* | 91 | 20.4 (8.4) | 20.8 (7.0) | 14.5 (7.2) | 13.7 (6.6) | 16.1 (7.4) | 14.6 (7.8) |
| *Year 3 or 4* | 58 | 19.3 (7.7) | 20.5 (8.7) | 16.2 (10.8) | 13.1 (6.8) | 15.2 (7.4) | 15.7 (10.9) |
| *p-value* |  | .79^+^ | .12^+^ | .22 | .80^+^ | .51^+^ | .81^+^ |
| **Relationship Status**^b^ | **275** |  | | | | | |
| *Married, Domestic Partner, Civil Union* | 96 | 20.2 (9.2) | 20.3 (7.8) | 16.6 (7.6) | 13.8 (6.7) | 14.4 (8.1) | 14.8 (8.1) |
| *Separated, Divorced, Widowed* | 179 | 20.0 (9.0) | 20.3 (8.9) | 15.7 (8.8) | 12.8 (6.4) | 16.9 (7.0) | 14.4 (8.7) |
| *p-value* |  | .43 | .48 | .20 | .14 | <.01 | .35 |
| **Race**^c^ | **257** |  | | | | | |
| *White* | 187 | 20.9 (9.6) | 20.2 (7.8) | 15.5 (8.1) | 12.9 (6.2) | 16.0 (7.6) | 14.7 (8.3) |
| *BIPOC* | 70 | 18.9 (7.5) | 20.2 (7.7) | 16.4 (9.2) | 13.2 (6.5) | 16.2 (7.2) | 15.1 (9.4) |
| *p-value* |  | .07 | .49 | .76^+^ | .35 | .42 | .99^+^ |
| **Gender identity**^d^ | **272** |  | | | | | |
| *Man* | 61 | 20.5 (12.5) | 19.2 (7.1) | 17.6 (10.4) | 14.1 (7.4) | 15.8 (8.3) | 12.8 (7.1) |
| *Woman* | 211 | 20.0 (7.9) | 20.5 (8.8) | 15.5 (7.6) | 12.9 (6.2) | 16.0 (7.1) | 15.1 (8.8) |
| *p-value* |  | .60^+^ | .15 | .10^+^ | .43^+^ | .43 | .15^+^ |
| **First generation status**^e^ | **274** |  | | | | | |
| *Yes* | 64 | 21.1 (11.7) | 19.6 (7.1) | 17.3 (10.7) | 13.8 (6.2) | 14.4 (7.4) | 13.8 (7.3) |
| *No* | 210 | 19.7 (8.1) | 20.5 (8.9) | 15.6 (7.5) | 12.9 (6.6) | 16.5 (7.5) | 14.8 (8.8) |
| *p-value* |  | .15 | .24 | .07 | .17 | .02 | .21 |
| **Considered dropping out**^f^ | **275** |  | | | | | |
| *Yes* | 41 | 19.3 (8.1) | 19.7 (7.0) | 15.1 (7.7) | 13.2 (7.6) | 16.0 (8.7) | 16.7 (10.0) |
| *No* | 234 | 20.2 (9.2) | 20.4 (8.7) | 16.1 (8.5) | 13.2 (6.3) | 16.0 (7.3) | 14.1 (8.2) |
| *p-value* |  | .30 | .23 | .24 | .33^+^ | .47 | .04 |

^+^Indicates that nonparametric testing used (Kruskal-Wallis or Mann-Whitney U)

Abbreviations: BIPOC: Black, Indigenous, and People of Color

^a^Post-hoc testing with Bonferroni correction resulted in no significant difference between group means.

^c^Race “white” includes participants who selected only “white” and “BIPOC” includes any participant who selected White and another race or only another race(s). Race was asked in a ‘select all that apply’ format so total percentage equates to more than 100%.

^d^For analysis of gender, cases were recoded into male and female; participants selecting ‘other’ or ‘prefer to self-describe’ were recoded as missing.

^e^First generation status “yes” includes participants who have no parent(s) with a college degree.

^f^Includes participants who answered “yes” to the question “have you seriously considered dropping out of training the past 6 months.”

**Table 2: Correlation Analysis of the Self-Weighted Secure Flourish Index (SFI) Scores domain percentage weights with demographics and intrinsic factors (N=280)**

|  | | **Self-Weighted Secure Flourish Index Domain Percentage Weights** | | | | | | | | | | | |
| --- | --- | --- | --- | --- | --- | --- | --- | --- | --- | --- | --- | --- | --- |
|  |  | **Happiness and Life Satisfaction** | | **Physical and Mental Health** | | **Meaning and Purpose** | | **Character and Virtue** | | **Close Social Relationships** | | **Financial and Material Stability** | |
|  | **n** | ***r*** | ***p-value*** | ***r*** | ***p-value*** | ***r*** | ***p-value*** | ***r*** | ***p-value*** | ***r*** | ***p-value*** | ***r*** | ***p-value*** |
| **Age** | 269 | -.03 | .60^+^ | -.04 | .57^+^ | -.02 | .72^+^ | .06 | .34^+^ | -.20 | <.01^+^ | .09 | .15^+^ |
| **Number of Dependents** | 267 | -.15 | .01^+^ | .01 | .90^+^ | .02 | .75^+^ | .07 | .25^+^ | -.16 | .01^+^ | .17 | .01^+^ |
| **WellRx**^a^ | 280 | -.03 | .62^+^ | -.02 | .75^+^ | -.03 | .59^+^ | .02 | .75^+^ | -.06 | .33^+^ | .06 | .31^+^ |
| **Brief COPE Inventory** | | | | | | | | | | | | | |
| *Avoidant* | 264 | -.01 | .89 | -.01 | .89 | -.01 | .88 | .06 | .36 | -.001 | .98 | -.02 | .81 |
| *Emotion* | 259 | -.03 | .67 | -.07 | .29 | .03 | .66 | .15 | .02 | .04 | .50 | -.08 | .21 |
| *Problem* | 260 | -.04 | .56 | -.02 | .80 | .09 | .14 | .15 | .02 | -.04 | .58 | -.12 | .05 |
| **Short-GRIT** | 257 | -.03 | .59 | -.09 | .17 | .12 | .06 | .10 | .11 | .05 | .45 | -.11 | .09 |
| **2QMBI**^b^ | 275 | .10 | .10 | -.03 | .65 | -.09 | .15 | .01 | .83 | -.11 | .06 | .10 | .11 |
| **DUREL** | | | | | | | | | | | | | |
| *Organized Religion* | 262 | -.16 | .01^+^ | -.14 | .02^+^ | .18 | <.01^+^ | .24 | <.001^+^ | -.03 | .67^+^ | -.07 | .23^+^ |
| *Nonorganized*  *Religion* | 262 | -.13 | .04^+^ | -.15 | .02^+^ | .19 | <.01^+^ | .28 | <.001^+^ | -.09 | .13^+^ | -.11 | .09^+^ |
| *Intrinsic Spirituality* | 258 | -.13 | .04 | -.10 | .12 | .20 | <.01 | .32 | <.001 | -.11 | .07 | -.11 | .08 |
| **Percentage Loans**^c^ | | | | | | | | | | | | | |
| *Education* | 271 | .07 | .22 | -.01 | .87 | -.06 | .32 | .11 | .07 | -.05 | .40 | -.05 | .44 |
| *Living* | 272 | -.01 | .87^+^ | -.03 | .67^+^ | .02 | .79^+^ | .02 | .78^+^ | .17 | <.01^+^ | -.04 | .52^+^ |

Abbreviations: 2QMBI: 2 question Maslach Burnout Inventory; DUREL: Duke University Religion Index

^+^Indicates that nonparametric testing used (Spearman Correlation)

^a^One question was added to the WellRx asking about access to physical and mental health care to meet the Social Determinants of Health domain of health care access and quality. One question on access to education was omitted as all students are currently enrolled in a graduate or doctoral program.

^b^The two questions in the 2QMBI were adjusted to fit the sample population by changing the words “job” and “work” to “training.”

^c^Represents the percentage of total each education and living expenses paid for with student loans. Cases with total expense percentages > or <100% were not included.
