# Supplementary material for: Individual values, the social determinants of health, and flourishing among medical, physician assistant, and nurse practitioner students
Source: PLoS One. 2024 Sep 27;19(9):e0308884. doi: 10.1371/journal.pone.0308884 (PMC11432832; doi:10.1371/journal.pone.0308884)
Supplement: S1 File — This file contains the complete survey used in this study. (PDF) [file pone.0308884.s003.pdf]

1. Which clinical health profession training program are you currently enrolled in?
- ☐ Physician Assistant/Associate
- ☐ Nurse Practitioner (MSN NP, clinical-based DNP)
- ☐ Medical Doctor
- ☐ None of the above (e.g., leadership DNP, PhD, accelerated RN)

Below, you will find a series of statements and questions regarding various aspects of your life. Please choose a single answer per statement. Select the number 0 to 10 that best describes your feelings about the statement. Please note that the meanings of numbers 0 and 10 vary by question and explanations are included with each line.

1. Overall, how satisfied are you with life as a whole these days? [0-Not satisfied at all; 10-Completely satisfied]
2. In general, how happy or unhappy do you usually feel? [0-Extremely unhappy; 10-Extremely happy]
3. In general, how would you rate your physical health? [0-Poor; 10-Excellent]
4. In general, how would you rate your overall mental health? [0-Poor; 10-Excellent]
5. Overall, to what extend do you feel the things you do in your life are worthwhile? [-Not at all worthwhile; 10-Completely worthwhile]
6. I understand my purpose in life. [0-Strongly disagree; 10-Strongly agree]
7. I always act to promote good in all circumstances, even in difficult and challenging situations. [0-Not true of me; 10-Completely true of me]
8. I am always able to give up some happiness now for greater happiness later. [0-Not true of me; 10-Completely true of me]
9. I am content with my friendships and relationships. [0-Strongly disagree; 10-Strongly agree]
10. My relationships are as satisfying as I would want them to be. [0-Strongly disagree; 10-Strongly agree]
11. How often do you worry about being able to meet normal monthly living expenses? [0-Worry all of the time; 10-Do not ever worry]
12. How often do you worry about safety, food, or housing? [0-Worry all of the time; 10-Do not ever worry]

Below you will find a list of 6 areas of life. Please assign a percentage (0-100%) to each area indicating how important you believe the topic is to your ability to flourish in life. The larger the percentage assigned, the more important this is to your ability to flourish. The total percentage assigned should equal 100%. Please only use the digit, and do not include the "%" symbol. For example, for 20%, you would enter "20." If you would like to assign 0% to a category, please enter "0" rather than leaving the space blank.

Happiness and life satisfaction _____
Mental and physical health _____
Meaning and purpose _____
Character and virtue _____
Close social relationships _____
Financial and material stability _____

Please answer the following questions regarding access to various resources.

1. In the past 3 months, did you or others you live with eat smaller meals or skip meals because you didn't have money for food? Yes/No
2. Are you homeless or worried that you might be in the future? Yes/No
3. Do you have trouble paying for your utilities (gas, electricity, phone, or internet bills)? Yes/No
4. Do you have trouble finding or paying for a ride (transportation)? Yes/No
5. Do you need daycare, or better daycare, for your kids? Yes/No/I do not have children
6. Do you need to work during training to support personal and/or school-related expenses? Yes/No
   a. Please indicate how many hours per week on average you work outside of any current training responsibilities. For example, if you are required to complete clinical time as part of your training, do you include these hours. [0-100 hours]

b. Do you have difficulty working as much as you need to for your necessary expenses due to training requirements? Yes/No

c. How do you believe working affects, if at all, your academic performance? Improves academic performance/ No impact on academic performance/ Hinders academic performance

7. Do you need help finding a job or a better job? Yes/No
8. Are you concerned about someone in your home using drugs or alcohol? Yes/No
9. Do you feel unsafe in your daily life? Yes/No
10. Is anyone in your home threatening or abusing you? Yes/No
11. Do you have difficulty accessing the mental or physical health help you need? Yes/No

1. In your current program, what year of training are you in?
   a. <3 months
   b. 3 months – 1 year
   c. Year 2
   d. Year 3
   e. Year 4

2. Which of the following represents your current gender identity?
   a. Male
   b. Female
   c. Other/Prefer not to disclose

3. What is your current age?
4. What is your current relationship status?
   a. Single (never legally married)
   b. Married
   c. Domestic partner
   d. Divorced
   e. Separate, but still legally married
   f. Civil union
   g. Widowed
5. How many legal dependents do you have?
6. Which race(s) best describe yourself? Please select all that apply:
   a. American Indian or Alaskan Native
   b. Asian
   c. Black or African American
   d. Native Hawaiian or other Pacific Islander
   e. White
   f. Prefer not to answer
7. Approximately what percentage of your education (undergraduate through current training) has been/is funded by each of the following? Please only use the digit, and do not include the "%" symbol. For example, for 20%, you would enter "20." If you would like to assign 0% to a category, please enter "0" rather than leaving the space blank.
   a. Student loans
   b. Personal cash/savings (self or shared expenses with spouse)
   c. Family cash/savings (extended family such as parents, grandparents, etc)
   d. Scholarship/grants (not including Pell Grant)
   e. Pell Grant
   f. GI Bill
   g. Something else

If you selected "something else" or would like to share any additional details about your tuition funding sources or experience, please do so here: [free text]

8. Approximately what percentage of your current living expenses are funded by each of the following? Please only use the digit, and do not include the "%" symbol. For example, for 20%, you would enter "20." If you would like to assign 0% to a category, please enter "0" rather than leaving the space blank.

a. Student loans
b. Personal cash/savings (self or shared expenses with spouse)
c. Family cash/savings (extended family such as parents, grandparents, etc)
d. Scholarship/grants
e. Federal or state supported resources (housing choice voucher program, Supplemental Nutrition Assistance Program (SNAP), Women Infant Children program, etc
f. Something else

If you selected "something else" or would like to share any additional details about your funding for living expenses, please do so here: [free text]

9. Do either or both of your parents have an undergraduate degree or higher? [Yes, both do/Yes, one does/No, neither do]

10. Have you seriously considered dropping out of your current program within the past 6 months? Yes/No
11. If you selected yes, please explain what factors impacted this decision:
a. Personal mental health
b. Personal physical illness
c. Family stress
d. Financial stress
e. Difficulty of course work
f. Lack of connection to the program
g. Something else

If you selected "something else" or would like to share any other thoughts regarding considerations for leaving training, please do so here: [free text]

For the following two statements, mark the box that most accurately reflects your response:

	Every day	A few times a week	Once a week	A few times a month	Once a month or less	A few times a year or less	Never
I feel burned out from my training.	○	○	○	○	○	○	○
I have become more callous toward people since I began training.	○	○	○	○	○	○	○

There are many ways to try to deal with problems. These items ask what you've been doing to cope with stress in your life. Obviously, different people deal with things in different ways, but I'm interested in how you've tried to deal with it. Each item says something about a particular way of coping. I want to know to what extent you've been doing what the item says. How much or how frequently. Don't answer on the basis of whether it seems to be working or not-just whether or not you're doing it. Use these response choices. Try to rate each item separately in your mind from the others. Make your answers as true FOR YOU as you can.

	I haven't been doing this at all	I've been doing this a little bit	I've been doing this a medium amount	I've been doing this a lot
I've been turning to work or other activities to take my mind off things.	○	○	○	○
I've been concentrating my efforts on doing something about the situation I'm in.	○	○	○	○
I've been saying to myself "this isn't real."	○	○	○	○
I've been using alcohol or other drugs to make myself feel better.	○	○	○	○
I've been getting emotional support from others.	○	○	○	○
I've been giving up trying to deal with it.	○	○	○	○
I've been taking action to try to make the situation better.	○	○	○	○
I've been refusing to believe that it has happened.	○	○	○	○
I've been saying things to let my unpleasant feelings escape.	○	○	○	○
I've been getting help and advice from other people.	○	○	○	○
I've been using alcohol or other drugs to help me get through it.	○	○	○	○
I've been trying to see it in a different light, to make it seem more positive.	○	○	○	○
I've been criticizing myself.	○	○	○	○
I've been trying to come up with a strategy about what to do.	○	○	○	○
I've been getting comfort and understanding from someone.	○	○	○	○

I've been giving up the attempt to cope.	○	○	○	○
I've been looking for something good in what is happening.	○	○	○	○
I've been making jokes about it.	○	○	○	○
I've been doing something to think about it less, such as going to movies, watching TV, reading, daydreaming, sleeping, or shopping.	○	○	○	○
I've been accepting the reality of the fact that it has happened.	○	○	○	○
I've been expressing my negative feelings.	○	○	○	○
I've been trying to find comfort in my religion or spiritual beliefs.	○	○	○	○
I've been trying to get advice or help from other people about what to do.	○	○	○	○
I've been learning to live with it.	○	○	○	○
I've been thinking hard about what steps to take.	○	○	○	○
I've been blaming myself for things that happened.	○	○	○	○
I've been praying or meditating.	○	○	○	○
I've been making fun of the situation.	○	○	○	○

Please respond to the following 8 items. Be honest - there are no right or wrong answers!

	Very much like me	Mostly like me	Somewhat like me	Not much like me	Not like me at all
New ideas and projects sometimes distract me from previous ones.	○	○	○	○	○
Setbacks don't discourage me.	○	○	○	○	○
I have been obsessed with a certain idea or project for a short time but later lost interest.	○	○	○	○	○
I am a hard worker.	○	○	○	○	○
I often set a goal but later choose to pursue a different one.	○	○	○	○	○
I have difficulty maintaining my focus on projects that take more than a few months to complete.	○	○	○	○	○
I finish whatever I begin.	○	○	○	○	○
I am diligent.	○	○	○	○	○

The following section contains 3 statements about religious belief or experience. Please mark the extent to which each statement is true or not true for you.

	Definitely not true	Tends not to be true	Unsure	Tends to be true	Definitely true of me
In my life, I experience the presence of the Divine (i.e., God).	○	○	○	○	○
My religious beliefs are what really lies behind my whole approach to life.	○	○	○	○	○
I try hard to carry my religion over into all other dealings in life.	○	○	○	○	○

What are the top stressors in your life?

____

What are the primary ways that you cope with the stressors of school?

____

How has your training changed you as a person?

____

Please share anything else you would like regarding your experience in your current training program or your thoughts on student wellness and flourishing.

____

How often do you attend church or another religious meetings?

- ○ Never
- ○ Once a year or less
- ○ A few times a month
- ○ A few times a year
- ○ Once a week
- ○ More than once/week

How often do you spend time in private religious activities, such as prayer, meditation or Bible study?

- ○ Rarely or Never
- ○ A few times a month
- ○ Once a week
- ○ Two more more times/week
- ○ Daily
- ○ More than once a day
